# Prolonged length of stay and its associated factors at adult emergency department in amhara region comprehensive specialized hospitals, northwest Ethiopia

**DOI:** 10.1186/s12873-023-00804-y

**Published:** 2023-03-29

**Authors:** Asnake Gashaw Belayneh, Yemataw Zewdu Temachu, Mengistu Abebe Messelu, Mignote Hailu Gebrie

**Affiliations:** 1 Department of Emergency and Critical Care Nursing, College of Medicine and Health Sciences, Bahir Dar University, Bahir Da, Ethiopia; 2 Department of Emergency and Critical Care Nursing, College of Medicine and Health Sciences, University of Gondar, Gondar, Ethiopia; 3 Department of Nursing, College of Medicine and Health Sciences, Debre Markos University, Debre Markos, Ethiopia; 4 School of Nursing, College of Medicine and Health Sciences, University of Gondar, Gondar, Ethiopia

**Keywords:** Emergency department, Length of stay, Northwest Ethiopia

## Abstract

**Background:**

Prolonged length of stay at the emergency department interferes with the main goal of emergency care and results in adverse patient outcomes like nosocomial infection, dissatisfaction, morbidity, and mortality. Despite this, little is known about the length of stay and the factors that influence it in Ethiopia’s emergency department.

**Methods:**

An institution-based cross-sectional study was conducted on 495 patients admitted at Amhara region comprehensive specialized hospitals emergency department from May 14 to June 15/2022. A systematic random sampling was employed to select study participants. A pretested structured interview-based questionnaire was used to collect data by using Kobo toolbox software. SPSS version 25 was used for data analysis. Bi-variable logistic regression analysis was carried out to select variables with P-value < 0.25. The significance of association was interpreted using an Adjusted Odds Ratio with a 95% confidence interval. Variables with P-value < 0.05 in the multivariable logistic regression analysis were inferred to be significantly associated with length of stay.

**Result:**

Out of 512 enrolled participants, 495 were participated with a response rate of 96.7%. The prevalence of prolonged length of stay in the adult emergency department was 46.5% (95%CI: 42.1, 51.1). Lack of insurance (AOR: 2.11; 95% CI: 1.22, 3.65), non-communicative presentation (AOR: 1.98; 95% CI: 1.07, 3.68), delayed consultation (AOR: 9.5; 95% CI: 5.00, 18.03), overcrowding (AOR: 4.98; 95% CI: 2.13, 11.68), and shift change experience (AOR: 3.67; 95% CI: 1.30, 10.37) were significantly associated with prolonged length of stay.

**Conclusion:**

The result of this study is found to be high based on Ethiopian target emergency department patient length of stay. Lack of insurance, presentation without communication, delayed consultation, overcrowding, and shift change experience were significant factors for prolonged emergency department length of stay. Therefore, interventions like expansion of organizational setup are needed to decrease the length of stay to an acceptable level.

**Supplementary Information:**

The online version contains supplementary material available at 10.1186/s12873-023-00804-y.

## Introduction

Emergency Department Length of Stay (EDLOS) is the total length of time for all emergency cases starting from arrival at the Emergency Department (ED) to the time the patient leaves the department [1]. According to Ethiopian hospitals guideline, patients requiring emergency services should be kept at ED for a maximum of 24 h. Any patient who needs care for longer than 24 h should be transferred to ward for inpatient management [2, 3].

Globally, in the last two decades, there is an imbalance between emergency service supply and demand, due to the annual ED visits rapidly increasing with the growing population, which affects emergency care as well as the length of stay [4, 5]. According to previous studies, prolonged EDLOS was 4% in England, 72.5% in Botswana, and 91.5% in Southern Ethiopia, which is relatively highest in Lower Income Countries [6–8].

Based on previous studies older age, lack of insurance, night shift presentation, moderate acuity level, being non-trauma patients, lack of inpatient bed, Boarding, overcrowding, delayed consultation, and delayed investigation has been identified as factors significantly associated with prolonged EDLOS [8–12]. Even though, some factors are identified the prevalence is not reducing yet [8].

Prolonged stay of patients in the emergency department causes a significant burden on the health care systems. EDLOS is considered as an indicator of crowding which is a serious global health delivery problem that negatively affects the quality, efficiency, and effectiveness of emergency care. Crowding will also prolong waiting time which reduces the capability of the ED to provide immediate stabilization of emergency patients [13, 14].

Life-threatening illnesses and injuries result in millions of deaths each year due to a lack of timely identification of patients for basic life-saving treatments and delayed care [15]. Prolonged ED stay increases mortality by 15–30% and admitted patients held in the ED longer may die more frequently than those admitted quickly and patients will leave without being seen [16, 17]. Longer ED length of stay (LOS) may compromise the quality of care and delay the emergency evaluation of other patients [18, 19]. Long ED stay will also increase health care cost on patients or health care systems [20], inpatient length of stay, and the risk of nosocomial infections [18, 21].

In Africa, EDs are increasingly charged with caring for patients with acute illnesses and injuries [22]. In the past 20 years, emergency care development primarily occurred in high-income countries (HICs), but is still limited in lower and middle-income countries (LMICs) [13]. A systematic review of emergency care conducted on 59 LMICs indicates a relatively high patient load and mortality particularly in Sub-Saharan African countries [23]. The combined effect of high patient flow and urgency of care make ED an essential area of focus for research in LMICs including Ethiopia in the next 10 to 20 years [24].

World Health Organization (WHO) recognizes emergency care as an important part of universal health care crucial in achieving the Sustainable Development Goal three (SDG_3_) [25]. Despite many measures were taken like team triage, adding health professionals, and primary health care services, prolonged EDLOS is still remained a global challenge [26, 27]. Currently, the WHO, 72nd World Health Assembly (WHA), and African Federation of Emergency Medicine reached a consensus on advancing research in LMICs to ensure access and timely health care. Millions of deaths and long-term disabilities from acute illness and injuries could be prevented if emergency care service is well exist and patients accessed it timely. It is projected that up to 54% of annual deaths in LMICs could be addressed by improved emergency care. Hence, research is needed to identify strategies that optimize timely prevention and treatment of conditions [28–30].

Ethiopia had no organized initiative program to improve emergency care until the late 1990s [31]. Currently, EDLOS is considered as one of the key performance indicator of Ethiopian hospitals [3]. The Ethiopian Ministry of Health in collaboration of with WHO and 72nd WHA has made efforts to bring an improvement in emergency care through training of emergency professionals and infrastructure building to reduce the burden of ED as well as EDLOS [29, 32, 33]. Even though there is some improvement, it is limited to some areas of the country and much more work is needed to be done, including research in unaddressed areas. There is little known data about emergency care services as well as EDLOS in Ethiopia and some significant variables like insurance status, mobility status, boarding time and communication which were recommended by the previous investigators are not assessed in the previous studies [31]. Therefore, this study was aimed to assess the length of stay at adult emergency department and associated factors in Amhara region comprehensive specialized hospitals, Northwest Ethiopia. The result of this study will provide an input to design strategies needed to reduce EDLOS that may significantly reduce healthcare costs, patient dissatisfaction, morbidity, and mortality.

## Methods and materials

### Study design and setting

An institutional-based cross-sectional study was conducted at emergency department of four comprehensive specialized hospitals in Amhara region from May 14 to June 15/2022. Debre Tabor comprehensive specialized hospital is found in the South Gondar zone of Amhara region 670 KM from Addis Ababa serving more than 2.3 million people with 225 beds. The average monthly ED patient flow was 450 [34].

Debre Markos comprehensive specialized hospital is found in the East Gojjam Zone of Amhara region 300 km away from Addis Ababa serving more than 3.5 million people in its catchment area. It has more than 216 beds and 30 patient catchment ability adult ED. The average monthly ED patient flow was 1000 [35].

Tibebe Ghion and Felege Hiwot comprehensive specialized hospitals are found in Bahir Dar, the capital of Amhara Regional State, which is 565 KM away from Addis Ababa serving more than 5 million people each with more than 500 beds. The average monthly ED patient flow was 1100 and 1050 respectively [36, 37].

## Participants of the study

All adult patients who were admitted to the emergency department of Amhara region comprehensive specialized hospitals were the source population. Those adult patients aged ≥ 18 who were admitted to the emergency department of the selected comprehensive specialized hospitals in Amhara region during the study period were the study population. Patients who went against medical advice, and patients with death on arrival were excluded from the study.

### Sample size determination

The Sample size was calculated by using Epi info Software version 7.2.4 by considering different factors which have a significant association in the previous studies such as lack of inpatient bed, overcrowding, and delayed investigation [8]. Then, the variable having the greatest value was chosen. Power of 80%, margin of error 5% and 95% confidence interval was taken into account. Finally, a 10% non-response rate was added and the final sample size used for the study was 512 (Table S1).

## Sampling procedure and technique

There are eight comprehensive specialized hospitals in Amhara region. From those, four hospitals were selected for this study by simple random sampling. In a month, an average of 3600 patients visit the emergency department of these four hospitals. The sample size was proportionally allocated to each respective hospitals based on their population size. Finally, a systematic random sampling technique was used to select a sample of 512 participants from each hospital with respective sampling intervals. The first participant was chosen randomly by lottery method. Then, other respondents were chosen at regular intervals by the data collectors until the required sample is completed.

## Operational definition

### Emergency department length of stay (EDLOS)

The period from the time of arrival to ED to the time of ED discharge/departure, which is calculated as the difference between the inflow time and the outflow time [1, 9].

### Prolonged emergency department length of stay

when a patient is kept at emergency department for more than 24 h/not disposed within 24 h [2, 8].

### Boarding

holding a patient in emergency department more than 4 h after the decision to admit or transfer has been made [38, 39].

### Overcrowding

A need for emergency services exceeds available resources in the ED, can be calculated as the proportion of patients staying at areas other than the treatment spaces / total patients in the ED *100, which should not be greater than 10% [40, 41].

### Triage time

is the time from patient arrived to ED until seen by the triage officer which should not exceed 5 min of arrival to ED [2].

**Waiting room time -** Time from triage until seen by the emergency clinician ranges from 0 min to 4 h for emergent and non-urgent patients based on South African Triage System/scale (SATS) [7, 18].

### Communication

Information exchange (liaison call before presentation) among ED of hospitals, dispatch center, prehospital care providers, other health facilities, or community with direct telephone or other services [2].

### Comorbidity

The coexistence of two or more conditions/cases in the same individual/patient [42].

### Consultation

Seeking assistance from another health care professional for therapeutic interventions, or other services that may benefit the patient, which should not be beyond 2 h in ED [43].

### Shift change experience

When the emergency health care professionals change their shift before the patient leaves the emergency department [44].

### Prehospital care

Out-of-hospital emergency care delivered by a bystander or trained health professional at least with transportation [45].

## Data collection tool and procedure

The data was collected from the adult emergency patients using an interviewer-administered structured questionnaire, patient chart review, from Health professionals, and by observation. The questionnaire was adapted from different guidelines and literatures [7–9, 12, 18, 46–49]. The questionnaire has four parts with a total of 48 questions including socio-demographic, presentation-related questions, clinical-related and institution-related questions. The data was collected by four trained BSc Emergency and Critical Care Nurses using Kobo toolbox software version 2022.1.2. Two MSc Emergency and Critical Care Nurses supervise the data collection process. The data was collected in a 24-hour period in two shifts. At first, eligible patients were identified by data collectors at the triage room while the triage nurse is triaging patients on arrival. At this point, socio-demographic characteristics and presentation-related characteristics were recorded. Then, other characteristics like investigations and overall therapies were obtained at different treatment points. Finally, the overall length of stay at the emergency department was documented shortly before the patient left the department.

## Data quality control

Data collectors and supervisors were trained for one day before data collection about the concept of the questionnaire, the required ethical conduct, the secrecy of the information, the rights of the participants, and the Kobo toolbox software to ensure consistency and to reduce variations between data collectors. There was daily communication between the principal investigator, supervisors, and the data collectors throughout the data collection process. The questionnaire was reviewed by a senior emergency clinician for face validity. A pre-test was conducted on 5% of the participants and necessary modification of the questionnaire was done based on the pre-test result before the actual data collection period. The data was cleaned and checked for completeness before analysis.

## Data processing and analysis

SPSS version 25 software was used for statistical analysis. Descriptive statistics including, frequencies and median with interquartile range (IQR) was computed. Multicollinearity was checked (Variance inflation factor = 2.04) and the model fitness was tested (p = 0.81) by using the Hosmer–Lemeshow goodness of fit test. Both bi-variable and multivariable logistic regression analysis was used to identify associated factors. Variables fulfilled P-value < 0.25 were entered into the multivariable logistic regression analysis model to control the effect of different confounding factors while assessing the effect of each independent variable with the outcome variable. The strength of the association was interpreted by using Adjusted Odds Ratio (AOR) with 95% Confidence Interval (CI). In multivariable analysis P-value less than 0.05 was declared as statistically significant with prolonged length of stay.

## Result

### Socio-demographic characteristics of study participants

Out of the total 512 recruited study participants, 495 were participated yielding a response rate of 96.7%. The median age of the participants was 38 with an interquartile range (IQR: 26–55) years. More than half (57.2%) of the study participants were males and two-thirds (66.9%) of the participants had health insurance support (Table 1).

**Table 1:** Socio-demographic characteristics of adult patients attending ED of Amhara region comprehensive specialized hospitals, Northwest Ethiopia, 2022 (n = 495)

Variables	Category	Frequency	Percent
Age in years	18–44	295	59.6%
	45–64	123	24.8%
	≥ 65	77	15.6%
Sex	Male	283	57.2%
	Female	212	42.8%
Place of residence	Rural	309	62.4%
	Urban	186	37.6%
Educational status	Unable to read and write	164	33.1%
	Able to read and write	93	18.8%
	Primary education	45	9.1%
	Secondary education	94	19.0%
	Higher education	99	20.0%
Occupational status	Employed	308	61.6%
	Unemployed	190	38.4%
Health insurance coverage	Yes	331	66.9%
	No	164	33.1%

### Time and presentation-related characteristics

Of 495 participants, the majority (81%) were triaged within five minutes of arrival and arrived at day time to the ED (80%). Nearly three-fourths (72.1%) of the participants visited the ED on weekdays with a private taxi being the most common mode of transport (71.9%) (Table 2).

**Table 2:** Time and presentation-related characteristics of adult patients attending ED of Amhara region comprehensive specialized hospitals, Northwest Ethiopia, 2022 (n = 495)

Variables Category Frequency Percent			
Triage time in minutes	> 5	94	19.0%
	≤ 5	401	81.0%
Patient’s arrival time of the day	Day shift	396	80.0%
	Night shift	99	20.0%
Arrival day of the week to the ED	Weekday	357	72.1%
	Weekend	138	27.9%
Mode of arrival to the hospital	Ambulance	86	17.4%
	private taxi	356	71.9%
	private car	26	5.3%
	on foot/walk-in	25	5.1%
	Other^*^	2	0.4%
Source of referral	Self	168	33.9%
	Health center	84	17.0%
	Private health facility	27	5.5%
	Public hospital	216	43.6%
Admission status of the patient	New	440	88.9%
	Repeat	55	11.1%
Communication with liaison officer before presentation	Yes	101	20.4%
	No	394	79.6%
Prior treatment/pre-hospital care	Yes	283	57.2%
	No	212	42.8%
Intervention given to the patient at triage	Yes	137	27.7%
	No	358	72.3%

**Note** Others* (police car).

### Clinical-related characteristics

More than one-third (39.2%) of the participants were in the orange triage category and more than three-fourths (78.2%) of the patients were alert. Regarding mobility one-fourth (26.7%) of the participants were moved with a stretcher/wheelchair (Table 3).

**Table 3:** Clinical-related characteristics of adult patients attending ED of Amhara region comprehensive specialized hospitals, Northwest Ethiopia, 2022 (n = 495)

Variables Category Frequency Percent				
Triage category of the patient		Red	79	16.0%
		Orange	194	39.2%
		Yellow	177	35.8%
		Green	45	9.1%
Mental status of the patient		Alert	387	78.2%
		Respond to voice	67	13.5%
		Respond to pain	25	5.1%
		Unresponsive/coma	16	3.2%
Mobility status of the patient		With stretcher/ immobile	132	26.7%
		With help	210	42.4%
		Walking without help	153	30.9%
Type of case/presenting compliant		Trauma	121	24.4%
		Non-trauma	319	64.4%
		Infectious	55	11.1%
Pre-existing comorbidity		Yes	125	25.3%
		No	370	74.7%
Cases per patient		1	370	74.7%
		≥ 2	125	25.3%
Pain duration in minutes		≤ 60	423	85.5%
		> 60	72	14.5%

### Institution-related characteristics

Almost half (46.7%) of the participants were examined within 10 min and almost two-thirds (61.2%) were evaluated by a medical intern initially. Almost three-fourths (73.5%) of the investigations and nearly two-thirds (63%) of the ordered drugs were available within the caregiving hospitals (Table S2).

## Emergency department length of stay

**Fig. 1:**
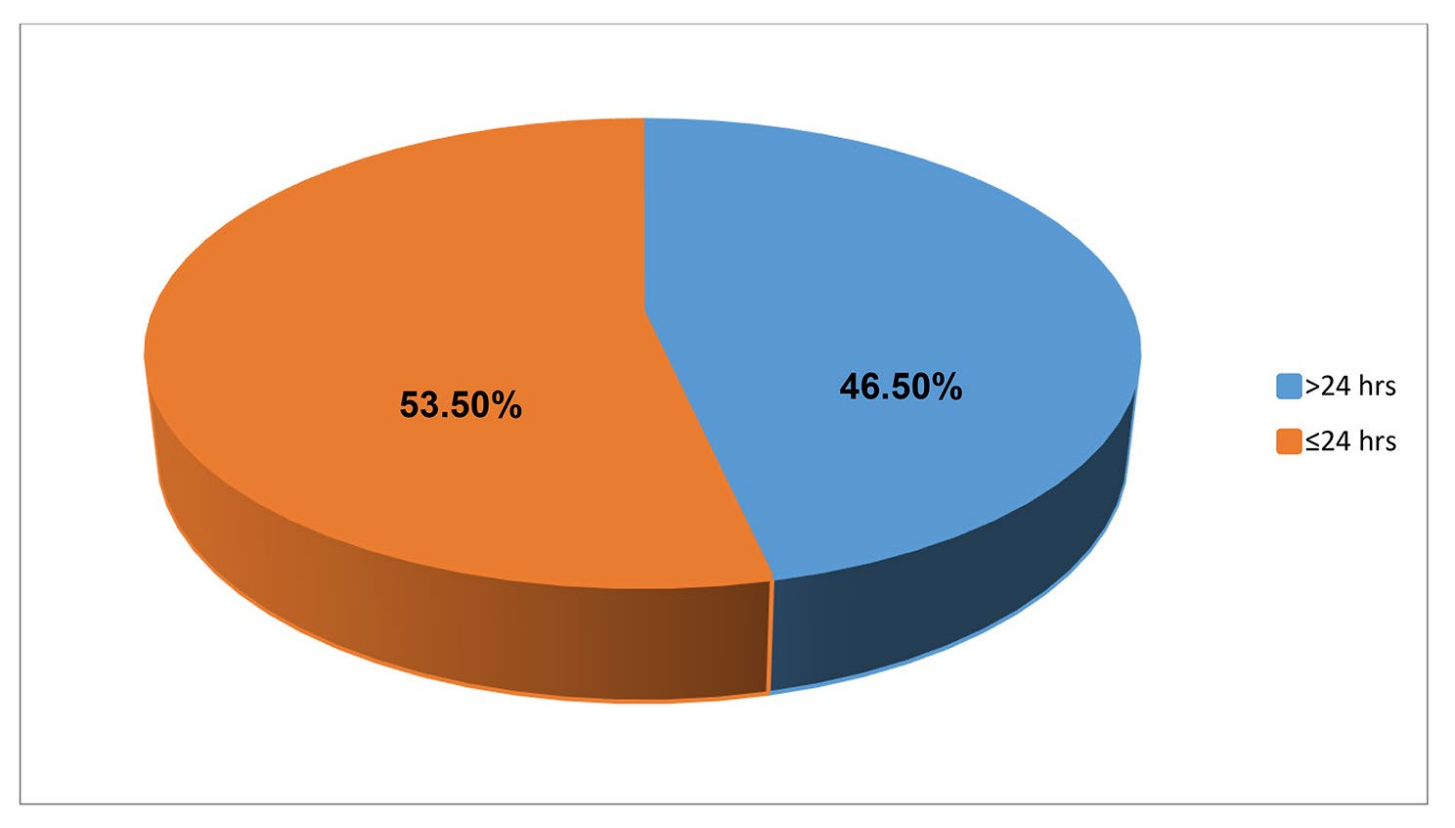
Proportion of emergency department length of stay in adult patients attending Amhara region comprehensive specialized hospitals, Northwest Ethiopia, 2022 (n = 495)

**Fig. 2:**
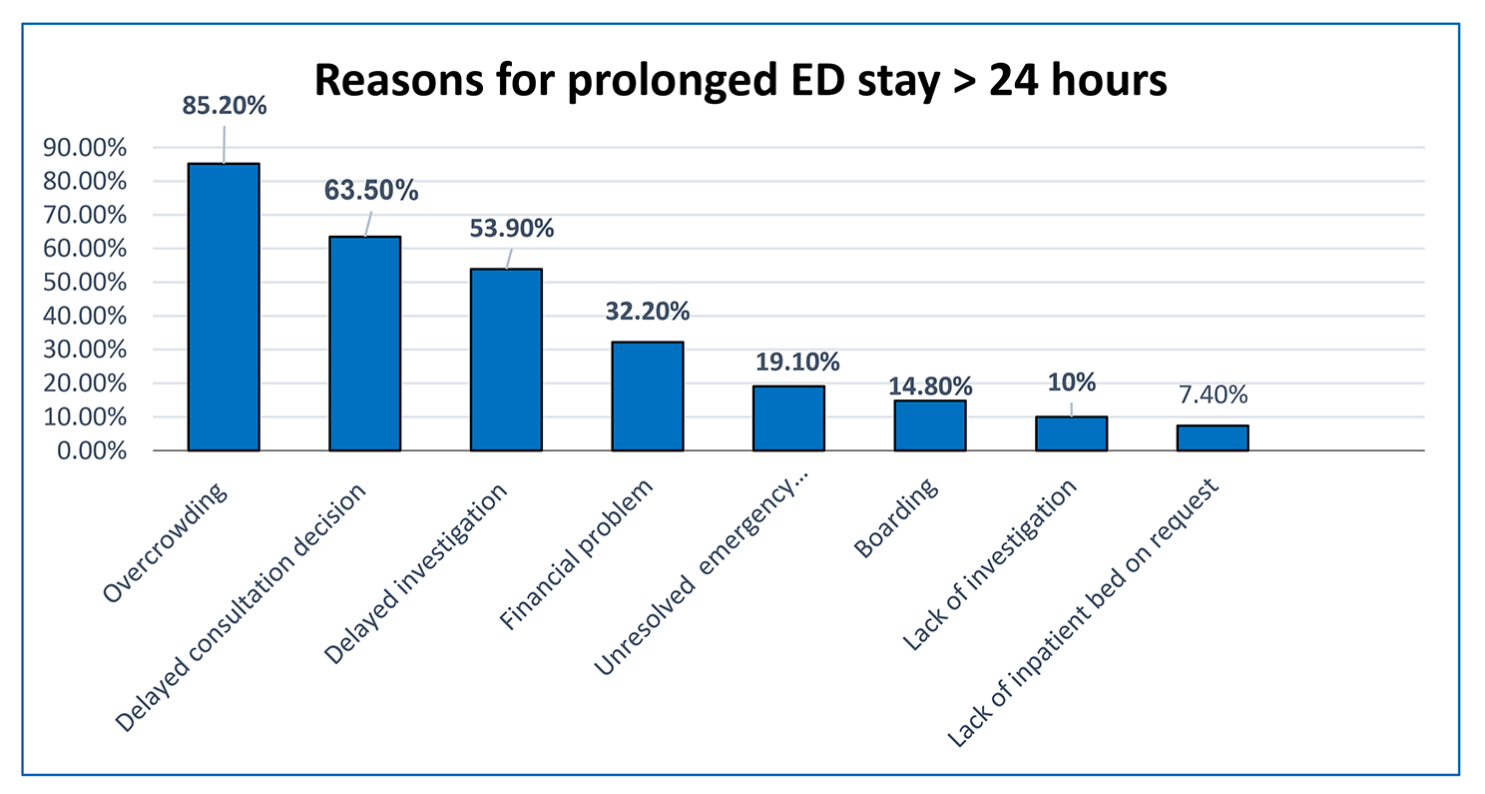
Reasons for prolonged ED stay in adult patients attending ED of Amhara region comprehensive specialized hospitals, Northwest Ethiopia, 2022 (n = 495)

Almost half 230 (46.5%) (95%CI: 42.1, 51.1) of the participants had prolonged (greater than 24 h) emergency department length of stay. The median length of stay was 23.1(IQR: 10.7–32) hours. The minimum and maximum ED stay were 1.6 and 171.5 h respectively (Fig. 1). The most common reason for prolonged emergency department length of stay was overcrowding (85%), followed by delayed consultation decision (63.5%) (Fig. 2).

### Factors associated with prolonged Emergency Department length of stay

In bi-variable logistic regression analysis, fourteen independent variables such as age, residence, education, health insurance, triage time, communication, comorbidity, number of cases per patient, type of examining clinician, duration of pain, number of consultation, consultation time, overcrowding, and shift change experience were associated with ED length of stay with P-value < 0.25 and become eligible for multivariable analysis.

In multivariable logistic regression analysis, health insurance, communication, consultation time, overcrowding, and shift change experience showed statistically significant association with ED length of stay. Accordingly, the odds of being stayed in the ED for more than 24 h were 2 times more likely (AOR: 2.11; 95% CI: 1.22–3.65) for patients who had no health insurance coverage compared to patients who had health insurance. Patients who presented without communication to the ED were 2 times more likely to have prolonged ED length of stay as compared to patients who presented with communication (AOR: 1.98; 95 CI: 1.07–3.68).

The likelihood of having prolonged ED length of stay among patients who experience delayed consultation on ED treatment were 9.5 times as compared to their counterparts (AOR: 9.5; 95% CI: 5.00-18.03). Patients with an overcrowded ED environment were five times more likely to have prolonged ED length of stay compared with their counterparts (AOR: 4.98; 95% CI: 2.13–11.68). Patients who experienced shift change of staffs were 3.67 times more likely to have prolonged ED length of stay as compared to patients without shift change experience (AOR: 3.67; 95% CI: 1.30-10.37) (Table 4).

**Table 4:** Bi-variable and multivariable logistic regression analysis of factors associated with ED length of stay in adult patients attending Amhara region comprehensive specialized hospitals, Northwest Ethiopia, 2022 (n = 495)

Variables	Length of stay		OR at 95% CI	
	> 24 h	≤ 24 h	COR (95% CI)	AOR (95%CI)
Age in years				
18–44	126	169	1	1
45–64	60	63	1.28(0.84,1.95)	1.10(0.56, 2.15)
≥ 65	44	33	1.78(1.07, 2.97)	1.25(0.55, 2.85)
**Residence place**				
Rural	155	154	1.49(1.03, 2.15)	1.47(0.70, 3.07)
Urban	75	111	1	1
**Educational status**				
Unable to read & write	80	84	1.53(0.92, 2.54)	0.62(0.22, 1.77)
Able to read & write	56	37	2.43(1.36, 4.34)	1.04(0.38, 2.81)
Primary education	22	23	1.54(0.75, 3.13)	1.48(0.49, 4.44)
Secondary education	34	60	0.91(0.51, 1.63)	0.94(0.41, 2.13)
Higher education	38	61	1	1
**Health insurance**				
Yes	134	197	1	1
No	96	68	2.08(1.42, 3.04)	2.11(1.22, 3.65)**
**Triage time in minute**				
> 5	54	40	1.73(1.09, 2.71)	1.55(0.79, 3.01)
≤ 5	176	225	1	
**Communication**				
Yes	37	64	1	1
No	193	201	1.66(1.06, 2.61)	1.98(1.07, 3.68)*
**Comorbidity**				
Yes	68	57	1.53(1.02, 2.30)	1.23(0.30, 5.11)
No	162	208	1	1
**Cases per patient**				
1	161	209	1	1
≥ 2	69	56	1.60(1.06, 2.41)	1.16(0.28, 4.83)
**Pain duration in minute**				
≤ 60	175	248	1	1
> 60	55	17	4.59(2.57, 8.17)	1.86(0.86, 3.86)
**Examining clinician**				
Intern	169	134	2.73(1.33, 5.62)	1.58(0.59, 4.23)
General practitioner	49	105	1.01(0.47, 2.17)	1.71(0.58, 4.98)
Resident	12	26	1	1
**N** **o** **of consultation**				
1	111	172	1	1
≥ 2	103	47	3.40(2.23, 5.17)	0.67(0.34, 1.33)
**Consultation time in hour**				
> 2	164	46	12.3(7.84, 19.4)	9.5(5.00, 18.03)**
≤ 2	50	173	1	1
**Overcrowding**				
Yes	218	177	9.03(4.80, 17.04)	4.98(2.13,11.68)**
No	12	88	1	1
**Shift change experience**				
Yes	223	204	9.53(4.23, 21.30)	3.67(1.30, 10.37)*
No	7	61	1	1

NB** indicates P-value < 0.01, * P-value < 0.05, and 1-reference.

## Discussion

Length of stay is a quality indicator in an emergency department used as a measure of ED performance in many countries of the world by allocating time targets based on specific ED populations and the level of health care system development [11, 47, 50]. This study aimed to assess the length of stay at adult emergency department and the factors associated with it. Accordingly, the prevalence of prolonged ED length of stay in this study was found to be 46.5% (95%CI: 42.1, 51.1), which means that almost half of the patients stay at emergency department more than 24 h. This finding is consistent with the previous studies conducted in Brazil (42.1%) and Malaysia (42.3%) [51, 52]. The possible reason may be due to similarity in study population and organizational structure. For example, in Brazil, all adult patients were included and 24 h used as a cut-point.

However, this finding is lower than the study conducted in Canada (58.6%), Australia (79.3%), Iran (61%), Botswana (72.5%), Addis Ababa-Ethiopia (84.3%), and Southern-Ethiopia (91.5%) [7, 8, 48, 53–55]. This discrepancy might be due to the difference in the study population, source of data, sample size, study period, and setting. The current study included all adult patients, but in Australia older adults > 65 years of age were included. Many studies mentioned that older age patients have long ED stays compared to other age groups [11, 56, 57]. In Canada and Addis Ababa-Ethiopia the study was done on patients requiring critical care admissions. Studies showed that patients requiring in patient admission have long ED stay than other ED patients [11, 12, 58–60].

In Iran, it may be due to the low sample size such that 77 participants were included, and the absence of triage. It may be also related to the time difference in year of study, since from the same study done in Iran in 2017 prolonged EDLOS was 10.2% [9]. In case of Botswana, it may be due to the difference in the source of data, since only the triage form was used as a source of data, which may not be complete and used to truly define the length of stay. In Southern Ethiopia, the study was conducted in a single hospital more than four years ago and the hospital was giving service over its capacity in which lack of inpatient bed (58.6%) was the major cause of prolonged EDLOS, but in this study, it is 7.4%. Moreover, currently, special attention is given for emergency and critical care development by Ethiopian Federal Ministry of Health [7, 8, 41].

On the other hand, this finding is higher than the study conducted in many European countries like France (15%), England (4%), Germany (13%), Norway (20.9%), and Netherlands,(13%), (38%), (16.3%) and (20%). This discrepancy might be due to the variation in the level of emergency health care system. As mentioned in the previous studies EDLOS is short in developed nations like Europeans which have developed emergency health care systems including specialized trauma and burn centers [6]. Whereas, in lower and middle-income countries there is limited care and it is the current growing focus area to deliver timely and qualified emergency care. Moreover, in Netherlands the study was done on trauma patients, in which studies showed that trauma patients have short ED stays than non-trauma patients [10, 13, 23, 32, 55, 61].

This study result is also higher than the study conducted in Asian countries like Iran (10.2%), Taiwan (8.2%) and (26.5%), China (41%), Israel (37.1%), Saudi Arabia (26.5%) and Jimma-Ethiopia. This discrepancy may be due to the difference in emergency setup, type of staff, study population, and sample size. For example, Iranian hospital is the most equipped hospital staffed with prehospital emergency team, experienced emergency specialists, and nurses [9]. But in this study except in some areas the emergency department is staffed with non-emergency professionals. Previous studies showed that evaluation by a medical student or non-trainee professional is associated with prolonged EDLOS [4]. In China and Saudi Arabia, the discrepancy may be due to sample size difference (4972) and (1360) participants were included in China and Saudi Arabia respectively [62, 63].

In Israel, the study was done on trauma patients [64], and in Taiwan, it was also done in a large hospital having 3600 beds with a specialized trauma center and on discharged patients in which trauma patients and discharged patients has short ED length of stay [12, 65]. Moreover, unlike many other countries patient in the Taiwanese ED usually seen by a physician shortly after triage. Because of easy medical evaluation access, patients do not wait a long time before being seen by an emergency physician. There is also a fast-track system for non-urgent medical patients [65–67]. In Jimma-Ethiopia, the discrepancy may be due to organizational structure and capacity. Jimma Medical Center has a well-developed emergency setup having greater than 65 emergency beds staffed with emergency physicians, emergency and trauma practitioner nurses. Previous studies showed that evaluation by trained ED professionals and specialists, availability of adequate inpatient bed, and organized emergency setup decrease EDLOS [4, 68].

The current study revealed that, patients without health insurance were 2 times more likely to have prolonged ED length of stay than those who do have it. This finding is in line with the study conducted in USA, Canada, and Iran [9, 57, 58, 69]. The possible reason may be due to the fact that having health insurance will decrease financial burden, especially for those with low-income status, and hence facilitate health care services in the hospital like investigation, drug, admission, and discharge process. But, for those who do not have, it may be a hindrance factor to accelerate service use and hence prolong EDLOS. Moreover, many uninsured patients use the ED for primary care with their less acute triage categories rather than clinics, hence they may wait for longer hours [9, 70].

This study revealed that patients who presented without communication to the ED were two times more likely to have prolonged EDLOS as compared to patients who presented with communication. This finding is supported by the study conducted in Israel [60]. The possible reason may be that, there will no emergency preparation for the new coming patient, bed will not be reserved and space will not available if a patient is presented without communication. Communication is important for early preparedness and response of emergency cases. Previous studies showed that communication and bed reservation decrease EDLOS for emergency cases [71].

This study found that, delayed consultation decision is more likely to increase EDLOS. This finding is in agreement with the study conducted in Netherlands [61], Iran[9], Indonesia [12], and Taiwan [65]. This can be explained that, as the amount of time taken to get services in ED increases, the EDLOS increases as well. Delayed consultation in ED can be caused by poor communication and insufficient number of consultants and this phenomenon found to increase EDLOS. Besides, junior doctors/Interns treat most of the patients and need more time to identify and consult the cases, so the decision-making process may be delayed [9, 72]. Moreover, conditions that need consultation are mostly severe cases and as mentioned in the previous studies patients with high and intermediate acuity level have the risk of prolonged EDLOS. Therefore, coordination and prioritization of service is important to manage accordingly [55, 73].

According to this study, crowding is significantly associated with prolonged EDLOS. This result is consistent with the study conducted in Sweden [47], Norway [40], USA [74], Italy [75], and Southern Ethiopia [8]. Many studies showed that crowding is a global challenge that affects emergency health care services and prolong EDLOS. As the number of patient who needs acute care rises, the supply and demand become mismatched that leads to overcrowding and patients may wait long times to get services, hence they may have prolonged EDLOS [40, 76].

This study also revealed that, Nurse-Physician shift change experience is significantly associated with prolonged EDLOS. This finding is supported by the study conducted in Israel [60], and Jimma-Ethiopia [46]. The possible reason may be explained that, during shift change, the emergency clinician may be tired or in a rush to leave the department rather than treating the patient and there will be a concurrent increase in workload that could increase LOS at these times. The handover period may preclude the service from responding to a new coming patient, while the out-going team handing over the previous patient. Responding to requests from primary teams, nursing staff, and patients requires that the new clinician review charts and records of all patients already on the service. Furthermore, the number of team members may be changed between shifts and the number of patients carried by a single clinician may increase, and this will increase the service burden. These all things may prolong EDLOS [44, 60, 77]. In this study, as a limitation there may be recall bias on the measurement of some variables like investigation and consultation time. Despite the limitation, this study is the first multicenter study in Ethiopia to provide information on adult patients’ emergency department length of stay.

## Conclusion

Based on the result of this study prolonged stay of adult patients at emergency department was found to be high based on Ethiopian target emergency department patient length of stay. Lack of health insurance, non-communicative presentation, delayed consultation, overcrowding, and shift change experience significantly associated with prolonged emergency department length of stay. Emphasis should be given to standardize emergency department and specialized units to enable timely access to care and stabilization. Expansion of community-based health insurance service to its target level will also be important to alieve the problem. Establishing a dispatch center will be also important to enable easy communication among hospitals. Applying fast track techniques for less urgent complaints and establishing onsite consultation services will decrease prolonged patient waiting time to get health care. Allocating enough time to hand over task during staff shift time will bridge gaps in patient care.

Note: Others* (police car).

## Electronic supplementary material

Below is the link to the electronic supplementary material.

Supplementary Material 1

## Data Availability

All data are available in the manuscript. Other supportive data will be available from the corresponding author on reasonable request.

## List of Abbreviations

AOR: Adjusted Odds Ratio
CI: Confidence Interval
COR: Crude Odds Ratio
ED: Emergency Department
EDLOS: Emergency Department Length of Stay
FMOH: Federal Ministry of Health
HIC: High Income Countries
IQR: Inter Quartile Range
LIC: Lower Income Countries
LOS: Length of Stay
NGO: Non-Governmental Organization
SATS: South African Triage System/Scale
SDG: Sustainable Development Goals
SPSS: Statistical Package for Service solution
USA: United States of America
WHA: World Health Assembly
WHO: World Health Organization
