# Supplementary material for: Prolonged length of stay and its associated factors at adult emergency department in amhara region comprehensive specialized hospitals, northwest Ethiopia
Source: BMC Emerg Med. 2023 Mar 29;23:34. doi: 10.1186/s12873-023-00804-y (PMC10053138; doi:10.1186/s12873-023-00804-y)
Supplement: Supplementary file 1 — Supplementary Material 1 [file 12873_2023_804_MOESM1_ESM.docx]

| **Variables** | **P_1_**(proportion of outcome among exposed) | **P_2_**(proportion of outcome among non- exposed) | COR | Power | 95% CI | Unexposed to exposed ratio | Sample size | | | Reference |
| --- | --- | --- | --- | --- | --- | --- | --- | --- | --- | --- |
|  |  |  |  |  |  |  | Exposed | Unexposed | Total |  |
| Crowding | 70.1 | 29.9 | 3.6 | 80% | 1.96 | 1:1 | 28 | 28 | 56 | **(**[**8**](#_ENREF_8)**)** |
| Lack of inpatient bed | 58.6 | 41.4 | 8.7 | 80% | 1.96 | 1:1 | 143 | 143 | 286 | **(**[**8**](#_ENREF_8)**)** |
| Delayed  Investigation | 56.7 | 43.3 | 3.7 | 80% | 1.96 | 1:1 | 233 | 233 | 466 | **(**[**8**](#_ENREF_8)**)** |

## Supplementary files

Table S1: Sample size determination to assess prolonged length of stay in adult emergency department of Amhara region comprehensive specialized hospitals, Northwest Ethiopia, 2022.

Table S2: Institution-related characteristics of adult patients attending ED of Amhara region comprehensive specialized hospitals, Northwest Ethiopia, 2022 (n=495).

| Variables | Category | Frequency | Percent |
| --- | --- | --- | --- |
| Waiting time in minutes | Immediate/0 | 5 | 1.0% |
|  | 1-10 | 231 | 46.7% |
|  | 10-60 | 228 | 46.1% |
|  | 60-240 | 31 | 6.3% |
| Type of clinician examined the patient | Intern | 303 | 61.2% |
|  | General practitioner | 154 | 31.1% |
|  | Resident | 38 | 7.7% |
| Type of investigation ordered | Blood test | 495 | 100.0% |
|  | Urine test | 139 | 28.1% |
|  | Stool test | 60 | 12.1% |
|  | Body fluid analysis | 13 | 2.6% |
|  | Electrocardiogram | 99 | 20.0% |
|  | Echocardiogram | 27 | 5.5% |
|  | X-ray | 165 | 33.3% |
|  | Ultrasound | 177 | 35.8% |
|  | CT-scan | 89 | 18.0% |
|  | Others^**^ | 14 | 2.8% |
| Number of investigations ordered | 1 | 25 | 5.1% |
|  | ≥2 | 470 | 94.9% |
|  |  |  |  |
| Did all ordered investigations available in the hospital | Yes | 364 | 73.5% |
|  | No | 131 | 26.5% |
|  |  |  |  |
| Time taken if all investigations available in hours | ≤2 | 205 | 56.3% |
|  | >2 | 159 | 43.7% |
|  |  |  |  |
| Time taken for partly available investigation in hours | ≤2 | 62 | 47.3% |
|  | >2 | 69 | 52.7% |

| Did all ordered drugs available in the hospital | Yes | 312 | 63% |
| --- | --- | --- | --- |
|  | No | 183 | 37% |
| Time taken if all drugs  Available in the hospital in hours | ≤1 | 161 | 51.6% |
|  | >1 | 151 | 48.4% |
| Time taken for partly available drugs in hours | ≤1 | 31 | 16.9% |
|  | >1 | 152 | 83.1% |
| Did the examining clinician request consultation | Yes | 433 | 87.5% |
|  | No | 62 | 12.5% |
| The number of consultation requested | 1  ≥2 | 283  150 | 65.4%  34.6% |
| Consultation time taken in hour | >2 | 210 | 48.5% |
|  | ≤2 | 223 | 51.5% |
| Emergency department overcrowded | Yes | 395 | 79.8% |
|  | No | 100 | 20.2% |
| The reasons for overcrowding | Patient overflow | 297 | 75.2% |
|  | Unlimited Patient relatives | 179 | 45.3% |
|  | Extra students | 23 | 5.8% |
|  | Delay in round | 106 | 26.8% |
|  | Insufficient bed | 42 | 10.6% |
|  | Boarding | 15 | 3.8% |
| Staff shift change experience | Yes | 427 | 86.3% |
|  | No | 68 | 13.7% |
| Patient disposition outcome | Discharged | 191 | 38.6% |
|  | Admitted | 280 | 56.6% |
|  | Referred | 15 | 3% |
|  | Died | 9 | 1.8% |
| Boarding time in hours | >4 | 37 | 7.5% |
|  | ≤4 | 458 | 92.5% |
| ED length of stay | >24 | 230 | 46.5% |
|  | ≤24 | 265 | 53.5% |

Note: - Others* (Cystourethrogram (CUG), Coronary angiogram, Magnetic Resonance Imaging (MRI), Endoscopy and sputum).
